# The Effect of Pertussis Vaccination During Pregnancy on the Binding Epitopes and Avidity of Anti–Pertussis Toxin Immunoglobulin G Antibodies in Infants and Their Mothers

**DOI:** 10.1093/infdis/jiag048

**Published:** 2026-01-28

**Authors:** Aapo Knuutila, Lauri Ivaska, Alex-Mikael Barkoff, Pieter van Gageldonk, Annemarie Buisman, Jussi Mertsola, Qiushui He

**Affiliations:** Research Centre for Infections and Immunity, Institute of Biomedicine, University of Turku, Turku, Finland; Department of Life Technologies, University of Turku, Turku, Finland; Department of Paediatric and Adolescent Medicine, Turku University Hospital, and University of Turku, Turku, Finland; InFLAMES Research Flagship Center, University of Turku, Turku, Finland; Research Centre for Infections and Immunity, Institute of Biomedicine, University of Turku, Turku, Finland; National Institute for Public Health and the Environment (RIVM), Centre for Infectious Disease Control, Bilthoven, the Netherlands; National Institute for Public Health and the Environment (RIVM), Centre for Infectious Disease Control, Bilthoven, the Netherlands; Department of Paediatric and Adolescent Medicine, Turku University Hospital, and University of Turku, Turku, Finland; Research Centre for Infections and Immunity, Institute of Biomedicine, University of Turku, Turku, Finland; InFLAMES Research Flagship Center, University of Turku, Turku, Finland

**Keywords:** immunization in pregnancy, vaccination, pertussis, pertussis toxin, infants

## Abstract

**Background:**

Immunization during pregnancy (IP) against pertussis protects young infants, but the maternally derived antibodies blunt the quantity of infants’ antibody responses to their primary vaccination. While the blunting effect has been well studied for antibody quantity, potential blunting that would affect functional characteristics of these antibodies is less studied. This study evaluated the effect of IP on the epitopes and avidity of anti–pertussis toxin (PT) immunoglobulin G (IgG) antibodies in infants and their mothers.

**Methods:**

In this prospective open-label controlled clinical trial, 47 pregnant women received diphtheria and tetanus toxoids and acellular pertussis (DTaP) vaccine booster, and 22 pregnant women who were not vaccinated served as controls. Sixty-nine infants received hexavalent DTaP vaccine at 3 and 5 months of age. The binding strength of anti-PT IgG antibodies and their ability to inhibit epitope-specific binding of mouse monoclonal antibodies were measured with enzyme-linked immunosorbent assays in both maternal and infant samples.

**Results:**

In both study groups, antibodies in cord blood showed higher epitope-specific inhibition and avidity than what was induced in infants after 2 primary vaccine doses, at age 6 months. Higher anti-PT IgG concentrations (*P* < .001) and epitope-specific inhibition targeting 1B7 (*P* = .049) and 11E6 (*P* = .02) were noted at age 6 months in control group infants, suggesting epitope-specific blunting in the IP group. No difference was observed in the avidity of anti-PT IgG at age 6 months between 2 study groups. The increase in avidity after vaccination was the highest in those mothers and infants with lower baseline avidity.

**Conclusions:**

IP decreased primary vaccination-induced antibody responses disproportionately against different PT epitopes in infants.

**Clinical Trials Registration:**

European Union Clinical Trial database (EudraCT no. 2019-001986-34; https://www.clinicaltrialsregister.eu).

## Teaser

IP with tetanus, diphtheria, and acellular pertussis vaccine resulted in anti–pertussis toxin immunoglobulin G transfer to infants with higher avidity and epitope-specific responses, suggesting good antibody-mediated protection. However, maternal antibodies blunted the infants’ epitope responses.

Acellular pertussis vaccines (aPVs) stimulate antibodies against pertussis toxin (PT), pertactin, filamentous hemagglutinin, and, with some formulations, fimbriae. While immunoglobulin G (IgG) antibody concentrations against aPV antigens are generally correlated with protection against pertussis [1–4], determining the exact protective levels has been challenging. Despite high vaccination coverage and booster doses administered to all age groups, *Bordetella pertussis* circulates in the population and infects vulnerable infants [5, 6]. The transplacental transfer of antibodies and immunization during pregnancy (IP) has been demonstrated to be effective in protecting infants less than 2 or 3 months of age (before they receive their own vaccines) against severe disease [7]. Maternal antibodies are transferred to the fetus via the placenta, with immunoglobulin G (IgG) being the only class that crosses effectively. IgG transport begins around the sixth week of pregnancy, increases gradually until week 28, and accelerates during the last trimester. Essentially, the earlier the vaccination, the lower the maternal antibody levels before the last trimester, when transfer is most efficient [8, 9]. However, studies show that IP decreases the quantity of the antibody response of infants to their primary pertussis vaccination [9–13].

Only limited data exist on the functional characteristics of the IP-induced and correspondingly blunted anti-pertussis antibodies in infants, such as their binding strength, binding epitopes, and neutralization efficiency. Antibodies targeting certain epitopes, such as the enzymatically active subunit (S) 1 targeting epitope 1B7 or cell receptor binding–related S2/3 specific epitope 11E6, are preferentially induced after infection more than with aPVs [14, 15] and have been demonstrated to be protective in animal models [16, 17]. Whether antibody responses to these potentially protective epitopes are modulated or blunted remains to be studied.

Regarding avidity, the multivalent binding strength between antibodies and the target antigen may demonstrate greater effectiveness at neutralizing the harmful effects of the toxin and can thereafter contribute to longer-lasting immunity against pertussis. Higher-avidity antibodies reflect B-cell maturation and the development of durable immunological memory [18], which in turn contributes to sustained protection against infection [19]. In the case of *Haemophilus influenzae* type b conjugate vaccines, rising avidity may help explain why strong protection is achieved even when circulating antibody levels are relatively low [18]. A similar pattern is seen with pertussis: elevated antibody titers alone do not reliably predict immunity, and some individuals remain protected despite modest concentrations [20, 21]. Earlier studies suggest that newborns of women immunized with tetanus, diphtheria, and acellular pertussis (Tdap) vaccine during pregnancy have greater avidity of umbilical cord IgG to PT than those born to unimmunized women [22]. However, blunting of the avidity development was observed at age 15 months after 4 primary doses of vaccine in infants born to vaccinated mothers [23].

The current study aimed to investigate the functional antibody characteristics before infants receive their own vaccines after IP. In addition, the effects of IP and existing immunity on the development of avidity and the epitope specificity of anti-PT IgG antibodies were studied in both mothers and their infants after the first 2 vaccine doses, up to 6 months after delivery.

## METHODS

### Study Design, Participants, and Study Procedures

In total, 69 mother-infant pairs completed this prospective, interventional, open-label controlled clinical trial, of whom 47 mothers received the Tdap vaccine (Boostrix; GSK) vaccine at 30–35 weeks of pregnancy (IP group), containing PT, filamentous hemagglutinin, and pertactin [9]. Twenty-two mothers were not vaccinated during pregnancy and served as a control group. Serum samples from mothers were collected before vaccination and at 48 hours and 6 months after delivery. In addition, cord blood samples were collected at delivery. The infants received hexavalent diphtheria and tetanus toxoids and acellular pertussis (DTaP)–inactivated poliovirus–*H. influenzae* type b–hepatitis B vaccine (Infanrix Hexa; GSK) at 3 and 5 months of age. Serum samples were collected before the first pertussis vaccine at age 3 months and 1 month after 2 primary doses at age 6 months. Peripheral blood mononuclear cells for plasma B-cell enzyme-linked immunospot assay measurement were collected 7 days after the second vaccine dose at 5 months and 7 days of age. Anti-PT IgG and PT-neutralizing antibody concentrations, as well as memory B-cell counts against PT, including their methods, were reported in detail elsewhere [9].

The trial was registered in the European Union Clinical Trial database (EudraCT no. 2019-001986-34) and was approved by the Ethics Committee of the Hospital District of Southwest Finland (ETMK no. 67/1800/2019). Written informed consent was obtained from all participants and the parents of the infant participants.

This exploratory analysis does not represent the whole cohort, since the functional antibody assays performed required considerable quantities of antibodies, which were used as predefined inclusion criteria [13, 18]. The baseline characteristics of the study participants were reported elsewhere [11]. In particular, only a few mother-infant pairs at paired time points could be included for longitudinal analysis of epitopes.

### Epitope-Specific Anti-PT IgG Antibodies

**Figure 1.**
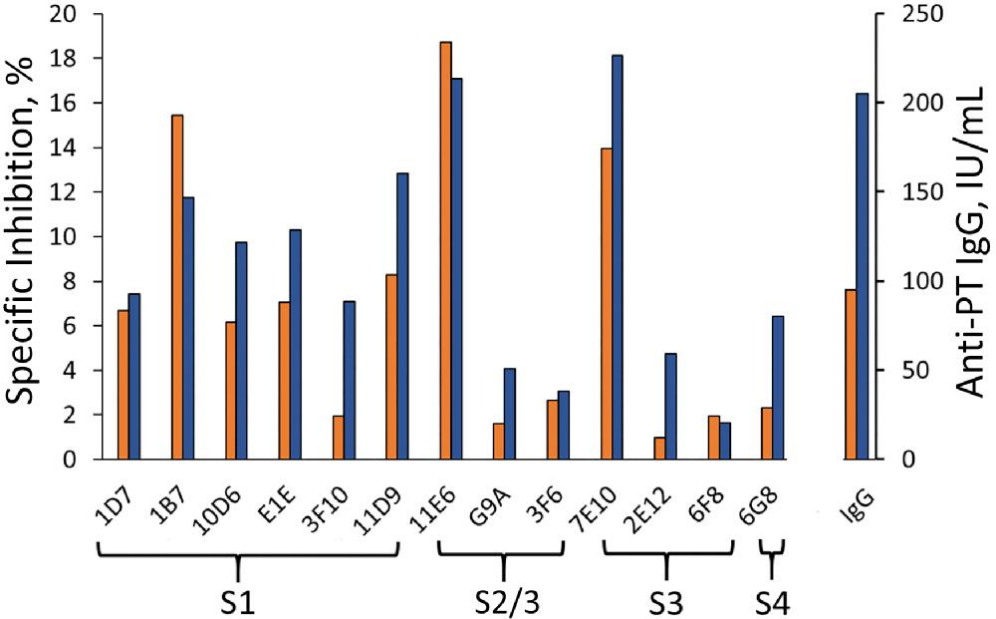
Overview of anti–pertussis toxin (PT) immunoglobulin G (IgG) and epitope-specific inhibition as geometric mean values of vaccinated mothers (n = 33) toward 13 epitopes (6 to subunit [S] 1, 3 to S2/3, 3 to S3, and 1 to S4) at 48 hours after delivery. The cases are differentiated between mothers with anti-PT IgG values <10 (*orange*; n = 17) or >10 (*blue*; n = 16) IU/mL before vaccination.

The binding sites of antibodies on native PT (GSK) were studied based on competition with murine monoclonal antibodies (mAbs) (National Institute for Biological Standards and Control). Serum samples from the mothers were tested for 13 epitopes (Figure 1), whereas the infants’ serum samples, owing to the limited amounts, were tested for epitopes 1B7 (S1), 11E6 (S2/3), and 7E10 (S3) [15, 16, 24].

The blocking of mAb binding to PT by serum antibodies was determined by enzyme-linked immunosorbent assay as described elsewhere [15], with the following modifications, which aimed to improve the measurement range of this assay to reliably estimate lower antibody levels. The serum samples (with >10 IU/mL of anti-PT IgG) were prepared in a 2-fold dilution series from 1:10 to 1:80 in 100 µL of 1% bovine serum albumin–phosphate-buffered saline (PBS) and incubated for 2 hours at 37°C. PBS was used as a control. However, the serum samples from the mothers were tested only at a dilution of 1:10 in duplicate to epitopes other than 1B7, 11E6, and 7E10. After the wells were washed with 0.9% sodium chloride–0.05% Tween-20 (C_58_H_114_O_26_), 100 µL of mAbs in PBS was added according to the dilutions recommended by the National Institute for Biological Standards and Control.

The duration of *P*-nitrophenylphosphate (O_2_NC_6_H_4_OP(O)(ONa)_2_.6H_2_O) substrate (catalog no S0942; Sigma) mediated with anti-mouse alkaline phosphatase (AP124; Merck) reaction incubation was optimized separately for each epitope by aiming for an absorbance value of 1.5 attained from the sample including only PBS. The absorbance was measured at 405 nm with a Victor Nivo device (Perkin Elmer). The specific inhibition of the maximum signal of mAb binding caused by similar epitopelike antibodies from study serum samples was determined from background reduced signals as 1 – absorbance (sample)/absorbance (PBS). The serum dilution resulting in a 50% reduction of mouse IgG monoclonal binding, the “specific inhibitory value” (half-maximal inhibitory concentration [IC_50_]), were evaluated from the dilution series of serum samples, which are later reported as their reciprocal values (1/IC_50_).

### Anti-PT IgG Avidity

Serum samples (with >2 IU/mL anti-PT IgG) were diluted to a concentration of 0.025 IU of anti-PT IgG per well and tested as described elsewhere [25, 26]. The sample wells were treated with 100 µL of 6.5-mol/L urea (OC(NH_2_)_2_) or PBS for 15 minutes, and the avidity index was calculated from the background (1% bovine serum albumin–PBS) reduced absorbance values as the absorbance (urea well)/absorbance (PBS well). Any sample with a absorbance measured from the PBS well lower than that in the in-house anti-PT IgG negative control (0.1 IU/mL) was excluded from further analysis.

### Statistical Analysis

The analyses were performed with IBM SPSS Statistics for Windows software, version 28.0 (IBM). Based on an assumption of normal distribution by Shapiro-Wilk test, the differences in means between the groups at each time point were tested with *t* tests and Bonferroni correction, and longitudinal analysis was performed with paired *t* tests. The 3-month time point was used as the baseline for evaluating infants’ vaccine responses. To evaluate the effect of the mother's existing memory of vaccination antigens on maternal and infant antibody levels, an analysis was performed grouping individuals based on high and low baseline antibodies or their affinity. The correlations are reported as Pearson or Spearman correlation coefficients when applicable. Two-sided statistical significance was set at *P* < .05.

## RESULTS

### Epitope-Specific Anti-PT IgG Antibodies in Mothers 48 Hours After Delivery

A comprehensive anti-PT IgG epitope profile at 48 hours after delivery was examined in mothers who received the Tdap booster during pregnancy (n = 33) (Figure 1). Extensive inhibition of mAb binding by human anti-PT IgG was observed toward S1 epitopes, whereas the inhibition of mAbs targeting S2, S3, and S4 was notably low (on average <5%), except for 11E6 (S2/3) and 7E10 (S3). At 48 hours after delivery, mothers with higher prevaccination anti-PT IgG concentrations (>10 IU/mL) had more antibodies inhibiting the mAbs targeting 6G8 (S4), 3F10 (S1), G9A (S2/3), and 2E12 (S3) epitopes than mothers with lower anti-PT IgG concentrations before vaccination. The magnitude of all the epitope-inhibiting specific responses was strongly correlated with the anti-PT IgG concentration (Pearson *R* = 0.842).

### Epitope-Specific Anti-PT Immunoglobulins in Infants After 2 Primary Doses

**Figure 2.**
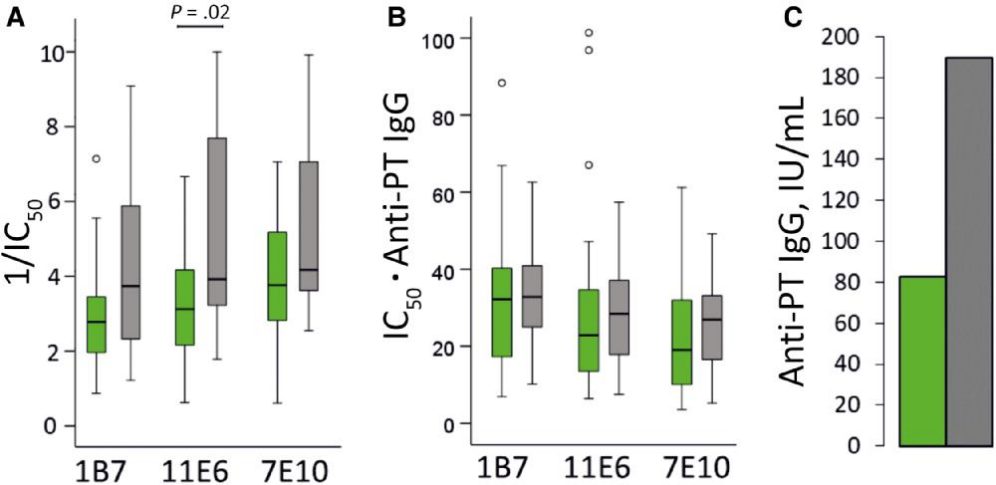
Epitope-specific monoclonal antibody inhibition among infants at 6 months of age in the immunization during pregnancy (IP) group (*green*; n = 27) and the control group (*gray*; n = 18). *A, B,* Box plots show the median, interquartile range (IQR), and 1.5 times the IQR of the 1/half-maximal inhibitory concentration (IC_50_) (*A*) and the IC_50_ values multiplied by the anti–pertussis toxin (PT) immunoglobulin G (IgG) concentrations (*B*) for epitopes 1B7 (subunit [S] 1), 11E (S2/3), and 7E10 (S3) at age 6 months. IC_50_ indicates the serum dilution at which half of the binding reactions are inhibited. The higher the reciprocal of IC_50_ (1/IC_50_ [*A*]), the more effective the serum was at blocking the binding of epitope-specific monoclonal antibodies. *C,* Geometric mean anti-PT IgG concentrations are presented for comparison. Open circles represent values exceeding 1.5 times the IQR. A sample in the IP group with an IC_50_ ⋅ anti-PT IgG value >250 in epitope 7E10 was excluded from the graph. The statistical significance of difference was assessed using *t* tests.

**Figure 3.**
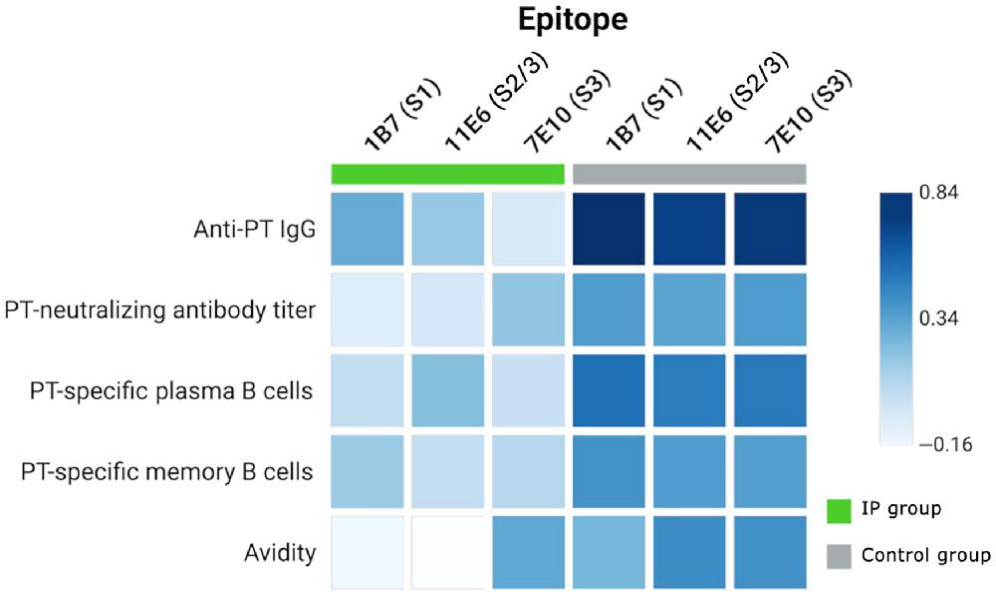
Correlation of epitope-specific monoclonal antibody inhibition and other immune responses in infants at age 6 months, presented as a heat map. Pertussis toxin (PT)–specific plasma B cells were measured 1 week after the second primary dose at age 5 months. Correlation was considered weak at *≤*±0.35, moderate between ±0.35 and ±0.75, and strong at ≥±0.75. Abbreviations: IgG, immunoglobulin G; IP, immunization during pregnancy. (Figure created using Biorender.)

At 6 age months, serum samples from infants in the control group had higher inhibition of all 3 epitope-specific mAb bindings than samples from infants in the IP group, born to mothers who had received the Tdap vaccine (Figure 2), statistical significance was noted only for 11E6 (*P* = .02). Epitope-specific antibodies were correlated moderately, with varying strengths, with avidity, the PT-neutralizing capability of antibodies, PT-specific plasma, and memory B cells in the control group but not in the IP group (Figure 3). Thereafter, a proportional analysis model was introduced to consider overall anti-PT IgG concentrations (Figure 2*B*). Based on this model, no difference was noted in the relative quantities of epitope-specific antibodies.

**Figure 4.**
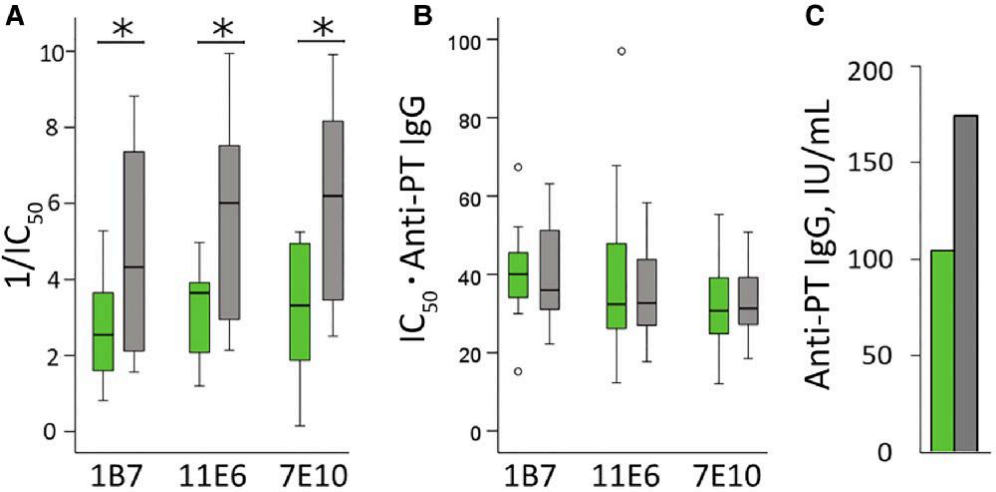
Samples obtained at 6 months of age were compared for epitope-specific antibody inhibition between infants in the immunization during pregnancy (IP) group (*green*; n = 12) and the control group (*gray*; n = 14); samples were compared only from infants with anti–pertussis toxin (PT) immunoglobulin G (IgG) values <10 IU/mL at age 3 months, before primary vaccination. *A, B,* Box plots show the median, interquartile range (IQR), and 1.5 times the IQR of the 1/half-maximal inhibitory concentration (IC_50_) (*A*) and the IC_50_ values multiplied by the anti-PT IgG concentrations (*B*). *C,* Geometric mean anti-PT IgG concentrations at age 6 months are presented for comparison. Open circles represent values >1.5 times the IQR. A sample in the IP group with an IC_50_ ⋅ anti-PT IgG value >250 in epitope 7E10 was excluded from the graph. *P < .05 (*t* test).

To assess whether blunting is due to IP and not just antibodies, epitope responses at age 6 months were compared between infants from the IP and control groups who had <10 IU/mL anti-PT IgG at age 3 months. Hypothetically, no blunting should occur without maternal antibodies [9]. Infants in the control group had, again, significantly higher epitope-specific inhibition to all 3 epitopes (Figure 4) and anti-PT IgG at age 6 months (*P* < .05). Considering anti-PT IgG concentrations , both groups had similar epitope profiles (Figure 4*B*).

Existing antibodies may influence the readout, as 11% of IP and 14% of control group mothers had anti-PT IgG levels >50 IU/mL before the study. On the contrary, 26% of IP group mothers had an increase of <25 IU/mL in anti-PT IgG at delivery compared with baseline. To pinpoint whether epitope responses are blunted due to the presence of maternal antibodies in general, not just IP, the infants were redistributed into groups based on having either <10 or >10 IU/mL anti-PT IgG at age 3 months, the criterion previously determined to best predict blunting of the infants’ anti-PT IgG responses [9]. High maternal IgG levels before vaccination blunted infant anti-PT IgG responses for 1B7 and 11E6 (*P* < .05) but not for 7E10.

### Paired Sample Analysis and Epitope-Specific Transfer to Infants

**Figure 5.**
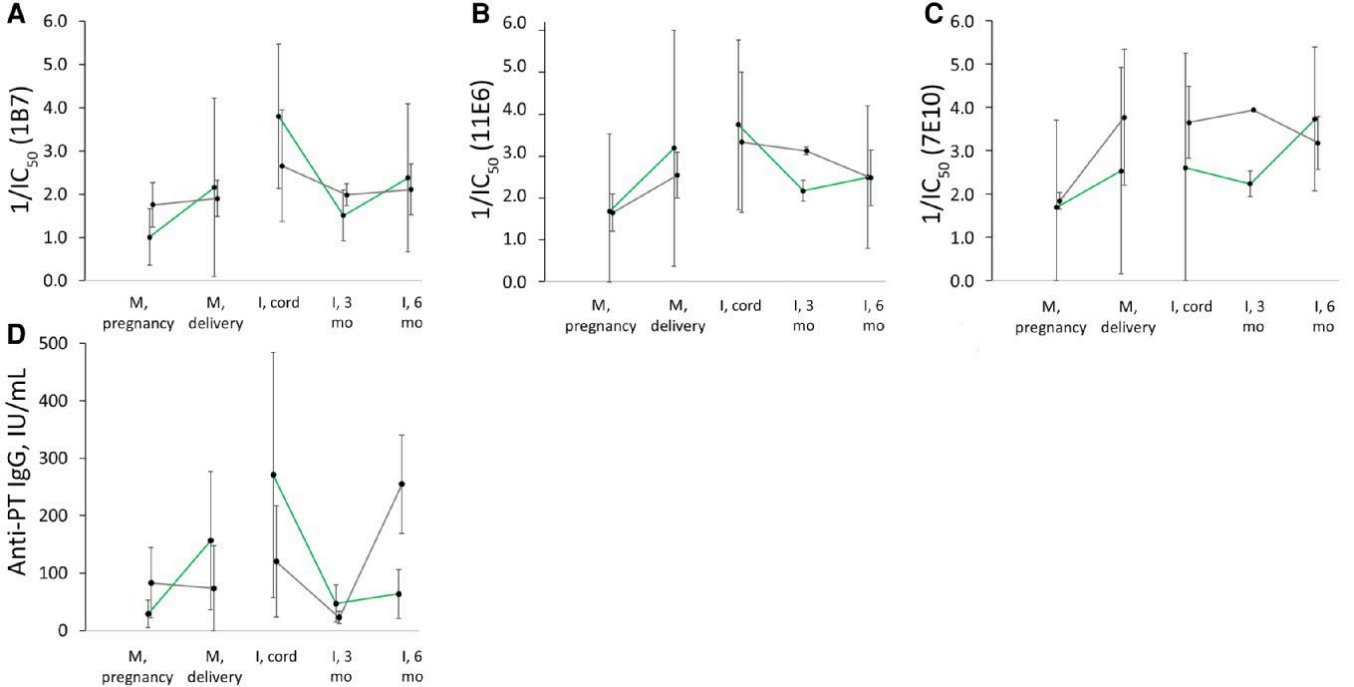
Transfer and development of pertussis toxin (PT)–specific antibodies inhibiting 3 epitopes (*A–C*), defined as geometric mean values of the 1/half-maximal inhibitory concentration (IC_50_), and anti-PT immunoglobulin G (IgG) values (*D*) in the immunization during pregnancy (IP) group (*green*; n = 11) and the control group (*gray*; n = 4). Only paired samples with anti-PT IgG values ≥10 IU/mL at all time points were included in the analysis. Abbreviations: I, infants; M, mothers.

Eleven IP and 4 control mother-infant pairs were studied for epitope-specific antibody transfer and the impact on infants’ responses. IP-induced antibodies inhibiting the 1B7, 11E6, and 7E10 epitopes were present in the infants’ cord blood and 3-month samples (Figure 5). Antibodies against 1B7 were more concentrated in the cord blood samples in both cohorts than in mothers’ 48 postdelivery samples but declined faster than 11E6 and 7E10 epitope-specific antibodies. Unspecific increases in 11E6 (S2/3) (*P* = .343) and 7E10 (S3) (*P* = .04) but not S1-targeting antibodies were detected 48 hours after delivery in all unvaccinated mothers (n = 4) included in this analysis. The mothers from the control group had very high concentrations of anti-PT IgG at the time of inclusion in the study (geometric mean, 83 IU/mL) but no increase in IgG between their visit during pregnancy and delivery (Figure 5*C*).

After 2 primary vaccine doses, there was an increase in the inhibition of epitope-specific mAbs only in IP group infants. However, there was no significant difference in the quantity of epitope-specific antibodies between the study groups at age 6 months, despite significantly lower anti-PT IgG concentration in the IP group (Figure 5*D*). The inhibition of epitope-specific mAbs at age 3 months was higher in the control group despite lower anti-PT IgG concentrations, which may reflect these observations. In relative terms, infants in control group induced a lot of anti-PT IgG after their first 2 vaccination doses, without a comparable increase in the measured inhibition toward the studied epitopes, but hypothetically with increases in inhibition to other epitopes.

### Avidity in Mothers

**Figure 6.**
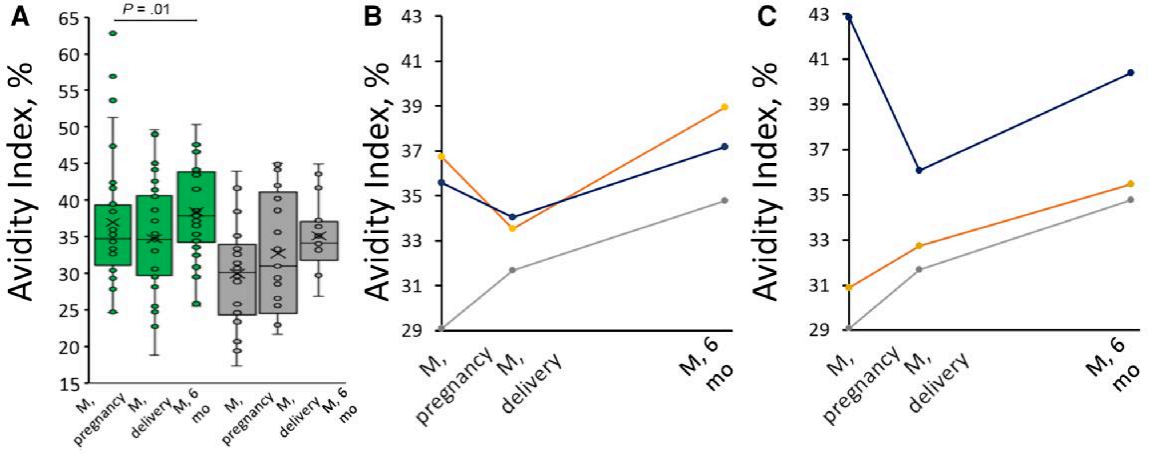
*A*, Mothers’ avidity index to pertussis toxin (PT) in the immunization during pregnancy (IP) group (*green*; n = 33) and the control group (*gray*; n = 18) before vaccination, at 48 hours after delivery, and at 6 months after delivery are presented as box plots; *X* within box plot represents the mean avidity index. *B, C,* Avidity development of mothers in the IP group based on lower (*orange*; n = 13) or higher (*blue*; n = 20) anti-PT immunoglobulin G (IgG) levels (<10 or ≥10 IU/mL, respectively) (*B*) or avidity index <35% (n = 17) or >35% (n = 16) (*C*) before vaccination. The control group is shown in gray (n = 18). A statistically significant difference by paired *t* test (*P* = .005) was noted at 6 months between the control group and the IP group subgroups with high prevaccine avidity and between the high- and low-avidity groups within the IP group (*P* = .02) (*C*). Abbreviation: M, mothers.

The avidity of anti-PT IgG in vaccinated mothers did not increase between the vaccination time point and the 48 hours after delivery time point, but a significant increase was noted between 48 hours after delivery and 6 months post partum (Figure 6; *P* = .01). A similar increasing trend in avidity was noted in the control group. This increase in avidity may be associated with a >80% general increase in anti-PT IgG geometric mean concentrations between 48 hours after delivery and 6 months after delivery in the control group. Mothers in the IP group with lower avidity to PT before vaccination had a greater increase in avidity at 6 months post partum, whereas a decrease was noted in mothers with higher prevaccine avidity (Figure 6*C*). Nevertheless, mothers with high avidity before vaccination had higher avidity 6 months after delivery (Pearson *R* = 0.566), despite a decrease. There was no difference in the development of avidity in mothers based on varying prevaccination IgG concentrations (Figure 6*B*). No correlation was found between the timing of delivery and the avidity of anti-PT IgG at any study time point in vaccinated mothers (Spearman *R* < ±0.27). No significant correlation was found between avidity and either anti-PT IgG or PT-neutralizing ability at any time point.

### Avidity in Infants

One month after receiving 2 primary vaccine doses, infants had weaker levels of anti-PT IgG avidity than those passed from the mother at birth and those at 3 months of age (Table 1). There was no significant difference between the study groups’ avidity at age 6 months (*P* = .09) or when infants were classified based on high or low prevaccine anti-PT IgG (*P* = .11) or avidity (*P* = .18). Similar to the vaccine responses during pregnancy in mothers (Figure 6), infants with the lowest avidity before vaccination showed the strongest increase in avidity after vaccination, whereas infants with high prevaccination avidity showed a strong decreasing trend, indicating a development trend of blunting for the avidity response (Table 1). No correlation was found between avidity and anti-PT IgG, PT-neutralizing ability, or plasma or PT-specific memory B cells at any time point in either study group.

**Table 1.** Avidity Index and Concentration of Anti–Pertussis Toxin Immunoglobulin G in Study Infants

Group	No. of Infants	Avidity Index (95% CI), %			Anti-PT IgG (95% CI), IU/mL		
		Cord	3 mo	6 mo	Cord	3 mo	6 mo
IP group	37	49.8 (46.8–52.7)	38.8 (35.9–41.8)	38 (36.0–40.0)	202.8 (141.9–263.7)	32.4 (22.2–42.7)	83.4 (67.0–99.8)
Control group	14	50.5 (47.3–53.7)	40.3 (36.5–44.1)	35.2 (33.2–37.1)	39.6 (9.7–69.6)	7.1 (2.7–11.5)	188.3 (128.0–248.5)
IP group by anti-PT IgG before vaccine							
<10 IU/mL	10	48.4 (41.4–55.3)	42.2 (35.4–49.1)	36.8 (32.6–41.0)	28.1 (16.0–40.2)	4.7 (3.1–6.4)	111.9 (78.7–145.1)
≥10 IU/mL	27	48.9 (45.3–52.6)	37.4 (34.0–40.9)	38.9 (36.0–41.7)	247.7 (172.1–323.4)	44 (30.5–57.5)	64.7 (48.6–80.7)
Control group by anti-PT IgG before vaccine							
<10 IU/mL	10	48.9 (44.3–53.5)	39.4 (35.0–43.9)	35.1 (32.1–38.1)	18.5 (10.2–26.9)	3.8 (2.1–5.6)	218.6 (109.6–327.7)
≥10 IU/mL	4	51 (46.1–55.8)	42.4 (30.1–54.7)	35.4 (28.4–42.3)	146.5 (0.1–300.5)	25 (6.1–43.9)	45.3 (14.3–77.8)
IP group by avidity before vaccine							
Low^a^	15	42.1 (37.8–46.4)	30.9 (27.9–33.8)	36.7 (32.3–41.2)	234.3 (135.5–333.2)	44.3 (25.2–63.4)	83.1 (50.9–115.3)
High^a^	22	53.4 (50.1–56.6)	44.1 (41.0–47.1)	39.4 (36.7–42.0)	157 (70.1–243.9)	25.9 (11.6–40.3)	73.6 (56.7–90.4)
Control group by avidity before vaccine							
Low^a^	8	48.2 (43.9–52.6)	35.8 (34.8–36.8)	34.8 (31.2–38.4)	49 (.1–119.5)	7.7 (.1–16.6)	113.4 (43.7–183.2)
High^a^	6	51.2 (44.8–57.6)	46.3 (40.2–52.4)	35.7 (31.4–40.0)	63.3 (.1–138.3)	12.8 (.1–26.6)	243.3 (38.3–447.8)

Abbreviations: CI, confidence interval; IgG, immunoglobulin G; IP, immunization during pregnancy; PT, pertussis toxin.

^a^Arbitrary cutoff for low/high avidity in infants defined as 38% at age 3 months.

## DISCUSSION

This study supports IP to ensure sufficient quantity and functionality of PT-specific antibodies for infants before completion of their primary vaccination series. Data show that maternally derived antibodies had higher inhibition toward key epitopes, higher affinity, and PT-neutralizing ability [9] than antibodies induced by infants. After 2 doses, infants maintain a high quantity of epitope-specific antibodies at 6 months.

Maternal antibodies significantly blunted infants’ epitope-specific antibody responses, similarly as with anti-PT IgG [9, 27]. The lack of correlation between avidity, neutralization ability, plasma, and memory B cells with the corresponding epitope-specific inhibition in infants born to vaccinated mothers is concerning, as antibodies targeting these epitopes (1B7 and 11E6) are protective in animal models [16, 17, 28]. PT stimulated fewer B cells than other vaccine antigens after 2 doses [9]; future work should assess correlations after the third dose.

Epitope-specific blunting largely disappeared between the study groups if the total anti-PT IgG concentrations were considered (Figures 2*B* and 4*B*), emphasizing that analysis models must consider multiple factors. Blunting was found to be not only antibody mediated; vaccination alone demonstrated reduced anti-PT IgG and epitope-specific responses. Maternal factors such as cytokines [29, 30], microbiome [31], and Toll-like receptor and HLA polymorphisms [32] may influence the outcome.

Maternal antibodies may cause blunting differently depending on PT epitope presentation from infections versus chemically detoxified vaccine PT [14, 15, 33]. Despite the low transfer of anti-PT IgG from unvaccinated mothers, epitope-specific antibodies appeared in high quantities and blunted the response toward these epitopes effectively in their infants (Figure 5). Hypothetically, antibodies induced by the infants target other epitopes, as these infants had a strong increase in overall anti-PT IgG. High inhibition of 11E6 and 7E10 epitopes in infants of unvaccinated mothers with high prestudy antibody levels suggests recent infections [15]. These maternal antibodies likely persist at 6 months.

Epitope-specific inhibitions after Tdap vaccination were higher in mothers with higher prevaccination anti-PT IgG, indicating stronger responses. Similarly, infants receiving 3 DTaP doses had higher inhibition of 11E6- and 7E10-specific mAbs than older children after a single booster, indicating benefits of existing memory [15]. Variation in maternal epitope responses may reflect differences in overall anti-PT IgG concentrations or epitope profiles between vaccinated and preexisting antibodies; for example, adults given 1 Tdap dose had less 1B7- and 11E6-specific inhibition than those recently infected [14]. Pertussis exposure may maintain higher epitope-specific antibodies and cell-mediated memory.

Avidity increased most in mothers with low prevaccination avidity from baseline to 6 months post partum. No correlation was found between delivery timing and avidity development in vaccinated mothers, nor with any of infants’ epitope responses. Previous studies report mixed results [34, 35]. The current study was not designed or powered to evaluate this aspect. Avidity in cord blood exceeded maternal that in postdelivery samples, consistent with preferential transport of high-avidity maternal antibodies across the placenta [36, 37]. Infants showed sharp avidity decline between birth and age 3 months, though the PT-neutralizing ability remained high [9]. Maternal antibody decay seems differential for avidity and neutralization ability. This remains hypothetical, as the half-life of antibodies is Fc receptor mediated [38]. Subclass degradation kinetics [39] and their selective transport [40–42] may also affect epitope-specific antibody persistence and infant vaccine responses (Figure 5).

Greater avidity in infants born to vaccinated mothers has been observed in cord blood, compared with controls [22, 43], though we observed no difference in avidity, only difference in quantity. Long-term, lower avidity occurs in infants after IP after a fourth vaccine dose at age 15 months, correlating with lower anti-PT IgG [23]. Avidity was higher after a third vaccine dose in infants of unvaccinated mothers than after a single booster or infection in older children [25], suggesting that >2 booster doses are needed for avidity maturation. At age 6 months, IP group infants had slightly higher avidity than controls, likely due to residual maternal high-avidity antibodies. Avidity increase after 2 doses occurred mainly in infants with baseline avidity regardless of concentration, similar to trends in older age groups [26]. Overall, avidity variation among infants was greater at 3 months than at 6 months of age.

This study has several limitations. First, strict exclusion criteria were applied to ensure reliable analysis, particularly ruling out samples with low anti-PT IgG. This reduced sample size, statistical power, and generalizability; results may not represent the entire cohort or the broader population. Second, additional cutoffs were introduced to examine the effect of natural boosting on mothers’ responses and in further blocking of infants’ responses [9]. For example, low/high prevaccine avidity thresholds were arbitrary and intended for balanced case distribution rather than indication of “bad” or “good” starting points. Previous studies have also included anti-PT IgG concentration as an exclusion criterion for avidity evaluation [43]. However, dilution to matching antibody concentrations demonstrated that existing IgG concentrations alone do not predict avidity or its development (Figure 6*B* and Table 1) [27]. The lack of strong associations between PT-specific avidity and epitope inhibition, as well as PT-neutralizing activity and B-cell counts, emphasize that these are individual characteristics requiring separate study [27]. Finally, this study focused solely on PT-specific responses, and extending similar methods to other vaccine antigens would be valuable.

In conclusion, maternal antibodies after IP exhibited greater inhibition to several epitopes and higher affinity than those induced in infants after 2 primary doses. Having existing memory to PT in mothers positively influenced the magnitude of epitope-specific antibodies, and their avidity transferred to the infants. This provides a reassuring rationale for IP, ensuring anti-PT IgG-mediated protection for infants before completion of their primary vaccinations. Maternal antibodies caused blunting, particularly toward 1B7 and 11E6 epitopes. However, the level of avidity remained unaffected until 6 months of age. The noted blunting and the lack of correlation between plasma and memory B cells and corresponding epitope binding in infants after IP are of potential concern. The results suggest that ≥2 doses of DTaP are beneficial and necessary for the development of infants’ functional antibody responses regarding target epitopes and avidity toward PT.

## Notes

***Acknowledgments.*** We thank Liisa Lund, University of Turku, for carrying out the laboratory tests. We thank Elisa Knuutila, Johanna Teräsjärvi, and Elina Tenhu, who were involved in the sample processing. The purified PT antigen was kindly provided by GSK, Belgium.

***Author contributions.*** Study conception and design: A. K. and Q. H. Data acquisition: A. K., L. I., A. M. B., P. v. G., A. B., J. M., and Q. H. Data analysis and interpretation: A. K. and Q. H., with contributions from L. I., A. M. B., and J. M. Writing of the first draft: A. K. and Q. H. Critical revision of the first draft: L. I., A. M. B., P. v. G., A. B., and J. M. All authors approved the content for publication and agreed to be available to ensure that questions related to the accuracy of any part of the work are appropriately investigated and resolved.

***Disclaimer.*** The supporters of this study did not have any role in the data collection, analysis, interpretation of the data, or the writing of the report.

***Data availability statement.*** The data underlying this article will be shared on reasonable request to the corresponding author.

***Financial support.*** This work was supported by the PERISCOPE (PERtussIS COrrelates of Protection Europe) project, which has received funding from the Innovative Medicines Initiative 2 Joint Undertaking (grant agreement 115910), and by the European Union's Horizon 2020 research and innovation program, the European Federation of Pharmaceutical Industries and Associations, the Bill & Melinda Gates Foundation, the Tampere Tuberculosis Foundation (grant 26006205 to Q. H.), and the Sigrid Juselius Foundation (grant 240045 to Q. H.).
